# A *Brucella melitensis* H38Δ*wbkF* rough mutant protects against *Brucella ovis* in rams

**DOI:** 10.1186/s13567-022-01034-z

**Published:** 2022-03-02

**Authors:** Pilar M. Muñoz, Raquel Conde-Álvarez, Sara Andrés-Barranco, María-Jesús de Miguel, Amaia Zúñiga-Ripa, Beatriz Aragón-Aranda, Miriam Salvador-Bescós, Estrella Martínez-Gómez, Maite Iriarte, Montserrat Barberán, Nieves Vizcaíno, Ignacio Moriyón, José M. Blasco

**Affiliations:** 1 Departamento de Ciencia Animal, Centro de Investigación y Tecnología Agroalimentaria de Aragón (CITA), Zaragoza, Spain; 2 Instituto Agroalimentario de Aragón-IA2 (CITA-Universidad de Zaragoza), Zaragoza, Spain; 3 Instituto de Salud Tropical, Instituto de Investigación Sanitaria de Navarra and Departamento de Microbiología y Parasitología, Universidad de Navarra, Pamplona, Spain; 4 Otology and Neurotology Group CTS495, Department of Genomic Medicine, GENYO Centre for Genomics and Oncological Research, Pfizer-University of Granada-Junta de Andalucía, Granada, Spain; 5 Departamento de Patología Animal, Universidad de Zaragoza, Zaragoza, Spain; 6 Departamento de Microbiología y Genética, Universidad de Salamanca, Salamanca, Spain

**Keywords:** Sheep, brucellosis, *Brucella*, *B. ovis*, rough, vaccine

## Abstract

*Brucella melitensis* and *Brucella ovis* are gram-negative pathogens of sheep that cause severe economic losses and, although *B. ovis* is non-zoonotic, *B. melitensis* is the main cause of human brucellosis. *B. melitensis* carries a smooth (S) lipopolysaccharide (LPS) with an N-formyl-perosamine O-polysaccharide (O-PS) that is absent in the rough LPS of *B. ovis*. Their control and eradication require vaccination, but *B. melitensis* Rev 1, the only vaccine available, triggers anti-O-PS antibodies that interfere in the S-brucellae serodiagnosis. Since eradication and serological surveillance of the zoonotic species are priorities, Rev 1 is banned once *B. melitensis* is eradicated or where it never existed, hampering *B. ovis* control and eradication. To develop a *B. ovis* specific vaccine, we investigated three *Brucella* live vaccine candidates lacking N-formyl-perosamine O-PS: Bov::CAΔ*wadB* (CO_2_-independent *B. ovis* with truncated LPS core oligosaccharide); Rev1::*wbdR*Δ*wbkC* (carrying N-acetylated O-PS); and H38Δ*wbkF* (*B. melitensis* rough mutant with intact LPS core). After confirming their attenuation and protection against *B. ovis* in mice, were tested in rams for efficacy. H38Δ*wbkF* yielded similar protection to Rev 1 against *B. ovis* but Bov::CAΔ*wadB* and Rev1::*wbdR*Δ*wbkC* conferred no or poor protection, respectively. All H38Δ*wbkF* vaccinated rams developed a protracted antibody response in ELISA and immunoprecipitation *B. ovis* diagnostic tests. In contrast, all remained negative in Rose Bengal and complement fixation tests used routinely for *B. melitensis* diagnosis, though some became positive in S-LPS ELISA owing to LPS core epitope reactivity. Thus, H38Δ*wbkF* is an interesting candidate for the immunoprophylaxis of *B. ovis* in *B. melitensis*-free areas.

**Supplementary Information:**

The online version contains supplementary material available at 10.1186/s13567-022-01034-z.

## Introduction

Brucellosis is a worldwide extended disease caused by gram-negative bacteria of the genus *Brucella* that has a severe impact on animal and human health [1]. The genus includes several species among which *B. melitensis* primarily produces brucellosis in sheep and goats and is the major cause of human brucellosis [2]. *B. melitensis* cells have a surface smooth (S)-type lipopolysaccharide (LPS) made of a lipid A-core oligosaccharide linked to an N-formylperosamine O-polysaccharide (O-PS) that carries the epitopes relevant in S *Brucella* serodiagnostic tests [3]. Sheep can also be infected by *B. ovis*, a species that displays a rough (R) LPS because it lacks a complete set of functional O-PS genes [4, 5]. Although non-zoonotic, *B. ovis* causes a serious disease manifested by genital lesions in rams and placentitis and abortions in ewes that result in important economic losses [6].

While some countries have eradicated *B. melitensis* through the combined use of vaccination and test and slaughter programs [7, 8], none has eradicated *B. ovis*. Attempts to eradicate *B. ovis* infection, sometimes misnamed ovine epididymitis, have often been focused exclusively on ram testing and culling, a strategy that has failed consistently because it overlooks the important epidemiological role of ewes [9–11]. Moreover, testing and culling becomes economically unfeasible when prevalence is moderate or high, making vaccination against *B. ovis* indispensable [11, 12]. Nevertheless, the only vaccine available for the immunoprophylaxis of *B. melitensis* and *B. ovis* is *B. melitensis* Rev 1, a live-attenuated S strain that elicits antibodies to the O-PS that interfere in *B. melitensis* serodiagnostic tests. Since these vaccinal antibodies encumber *B. melitensis* eradication and surveillance and this zoonotic species is a priority, Rev 1 is banned after *B. melitensis* eradication, and the same reasons make Rev 1 unsuitable for use in *B. melitensis*-free countries but yet affected by *B. ovis*. Hence, the development of *B. ovis*-specific vaccines is of great interest, as proven by the dramatic increase in *B. ovis* prevalence in European Union countries shortly after Rev 1 withdrawal [6, 13].

Altering the antigenic structure of S *Brucella* has been the preferred strategy to develop vaccines that circumvent the interference in S serological tests. Logically, O-PS removal was the first choice and some R mutants have been investigated as *B. ovis* vaccines. The empirically developed *B. abortus* RB51 (the only R vaccine currently marketed) does not confer adequate protection against either *B. melitensis* or *B. ovis* [14, 15]. However, experiments in mice show that genetically well-defined R vaccines are superior to RB51 and could be effectual against *B. ovis* [16]. An antigen removal strategy was also followed to obtain a *B. melitensis* Rev 1 lacking protein BP26 but, although effective against *B. ovis*, this Rev 1 derivative obviously triggers anti-O-PS antibodies and the associated BP26-iELISA is not sensitive enough to be used as differential test [17]. Following a positive tagging strategy, a Rev 1 vaccine carrying the green fluorescent protein (GFP) gene and its ancillary GFP-ELISA were investigated in sheep [18]. Yet, co-inoculation of the GFP-tagged vaccine with soluble GFP plus a GFP booster in Freund’s adjuvant ten weeks later are necessary to trigger GFP antibodies matching the persistence of O-PS antibodies, a protocol of no practical use. Finally, Rev1::*wbdR*Δ*wbkC*, a construct carrying *wbdR* (an *E. coli* LPS acetyl-transferase gene) instead of *wbk*C (the *Brucella* LPS formyl-transferase gene) conferred protection against *B. ovis* in mice and displayed a N-acetyl-perosamine O-PS structure triggering antibodies that could be differentiated from those induced by *B. melitensis* in this laboratory model [19].

Since it is a naturally R species, a homologous *B. ovis* vaccine could also be an alternative to Rev 1. Classical bacterins in adjuvant are unsatisfactory as *B. ovis* vaccines [10, 12] and, although adjuvant-encapsulated outer membrane-rich extracts are effective [20, 21], the complexity of their preparation and the need for revaccinations make these vaccines expensive and unpractical. Recently, a polymeric BLSOmp31 antigen in oil adjuvant has been proposed as a *B. ovis* vaccine but the vaccine did not prevent infection in rams [22, 23]. An alginate-encapsulated *B. ovis* mutant in a putative ABC transporter has been reported to confer perfect protection in rams [24, 25] but this claim was based on a deficient bacteriological search for the challenge strain as only minimal aliquots of diluted homogenates were cultured and some important target organs [26] were not examined. In mice, we have reported that Omp25d and Omp22 mutants, a triple Omp10-Omp31-SP41 mutant and mutants with a truncated LPS-core lateral branch are attenuated and protect against *B*. *ovis* [27–29]. In parallel, we have investigated *B. ovis* CO_2_-dependence, a characteristic of this species that would encumber a practical use of homologous vaccines. This trait relates to mutations in carbonic anhydrase (CA) genes and can be abrogated by insertion of CA homologues of CO_2_-independent brucellae without affecting virulence in mice [30, 31].

In this work, we pursued three of the above-summarized strategies to develop *B. ovis* vaccines minimizing the interference in O-PS serological tests. For the R vaccine approach, we investigated a *B. melitensis* H38 *wbkF* mutant carrying an R-LPS with intact core oligosaccharide. This choice was based on previous works that show that dysfunction of this gene in this strain, while not matching Rev 1, results in adequate attenuation and better protection against *B. melitensis* in sheep than that obtained with mutants in other LPS genes [32, 33]. In the Rev 1 background, we studied the above-described Rev1::*wbdR*Δ*wbkC*. Finally, we also examined a *B. ovis* CO_2-_independent construct deleted in *wadB*, a gene necessary for full assembly of the R-LPS core oligosaccharide lateral branch of brucellae.

## Materials and methods

### Bacterial strains, conservation and growth conditions

The parental strains used for the construction of the vaccine candidate mutants are described below. Bov::CA is a *B. ovis* PA derivative carrying the active carbonic anhydrase (CAI and CAII) genes of *B. abortus* 2308 W in a stable Tn7 insert in chromosome II that is CO_2_-independent and retains the virulence of the wild-type in the mouse model [30, 31]. *B. melitensis* H38 is a S virulent strain used previously in the analysis of LPS genes [32], and *B. melitensis* Rev 1 is the OIE recommended sheep brucellosis vaccine [34].

*B. ovis* PA (wild-type, virulent, serum and CO_2_-dependent) and its kanamycin-resistant virulent derivative (BoPA-Km^R^) have been respectively used as challenge strains in sheep and mice in previous works [19, 35].

All the above bacteria and the corresponding mutants (see below) were maintained freeze-dried or in skimmed milk at −80 °C in the CITA (Zaragoza, Spain) *Brucella* culture collection. To prepare the bacterial suspensions for the experiments described below, the stocks were rehydrated or thawed and seeded onto Blood Agar Base (BAB no. 2, OXOID, USA) or BAB with 5% sterile calf serum (BAB-S, for *B. ovis* PA) plates, for 3–4 days at 37 °C in air or, for *B. ovi*s PA, in 10% CO_2_ atmospheres.

### Construction and characteristics of vaccine candidates

The origin and characteristics of the primers and plasmids used in this work are presented in Additional files 1 and 2, respectively.

Bov::CAΔ*wadB* was constructed using the suicide plasmid pJQKΔ*wadB* (pYRI-2 in [36]), which was introduced into Bov::CA by conjugation with *E. coli* S17 λpir [37]. The first recombination event was selected by nalidixic and kanamycin resistance and the double recombination by nalidixic and sucrose resistance, and kanamycin sensitivity. Deletion of *wadB* was confirmed by PCR using oligonucleotides *wadB*-F1 and *wadB*-R4 (Additional file 1), which amplified a fragment of 570 base pairs (bp) in the mutant and a fragment of 1011 bp in the sibling revertant strain. Oligonucleotides *wadB-*F1 and *wadB*-R5 (Additional file 1), which amplify a fragment of 471 bp only in the wild-type strain, were used to verify the deletion.

*Brucella melitensis* H38Δ*wbkF* (R mutant in-frame deleted in the bactoprenol priming for O-PS polymerization gene; henceforth H38Δ*wbkF*) was constructed using the same methodology and genetic tools described for the homologous *B. suis* Bs2Δ*wbkF* mutant [38]. Briefly, pJQKΔ*wbkF* was transformed into competent *E. coli* S17 λpir (Additional file 2) and, after conjugation with *B. melitensis* H38, the first recombination event was selected by nalidixic acid and kanamycin resistance, and the double recombination by nalidixic and sucrose resistance, and kanamycin sensitivity. Deletion of *wbkF* was confirmed by PCR using oligonucleotides *wbkF*-F1 and *wbkF*-R4 (Additional file 1), which amplified a fragment of 953 bp in the mutant and a fragment of 1796 bp in the sibling revertant strain. Oligonucleotides *wbkF*-F1 and *wbkF*-R6 (Additional file 1), which amplified a fragment of 680 bp only in the wild-type strain, were used to verify the deletion.

Rev1::*wbdR*Δ*wbkC* construction and characterization has been described in a previous work [19]. Briefly, Rev1::*wbdR* was obtained inserting *wbdR* in chromosome II of *B. melitensis* Rev 1 using a Tn7 strategy [39]. Then, *wbkC* (formyltransferase gene) was deleted to obtain Rev1::*wbdR*Δ*wbkC,* which carries *N*-acetyl-perosamine instead of *N*-formyl-perosamine as proved by immunochemical and NMR analyses [39].

All vaccine candidates were stored at −80 °C in skimmed milk and grown as described above.

### Virulence and protection studies in mice

These experiments were carried out using seven-week-old female BALB/c mice (ENVIGO, Harlan) that were maintained in a BSL-3 facility (ES/31-2010-000132) with water and food ad libitum for 2 weeks before and during the experiments in accordance with the current European (directive 86/609/EEC) and Spanish (RD 53/2013) legislations.

To obtain inocula, fresh cultures (see “Bacterial strains, conservation and growth conditions”) were harvested in buffered saline solution (BSS; 0.015 M NaCl, 7 mM KH_2_PO_4_, 10 mM K_2_HPO_4_; pH 6.85) and suspensions adjusted spectrophotometrically (600 nm) to the appropriate dose (see below). Purity, absence of S-R dissociation (when appropriate) and exact doses (triplicate measurements) were assessed retrospectively following standard protocols [40].

**Figure 1:**
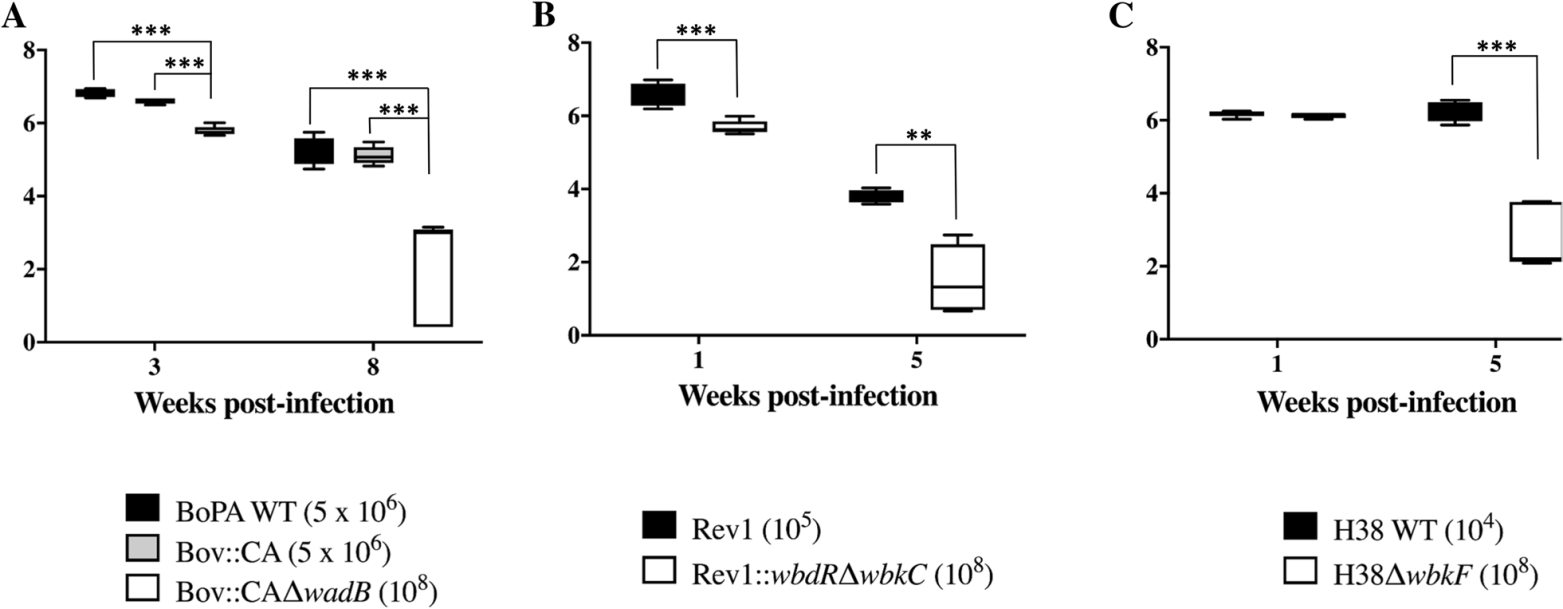
**Multiplication profiles in mice of the vaccine candidates Bov::CAΔ*wadB* (A), Rev1::*wbdR*Δ*wbkC* (B, taken from **[19]**) and H38Δ*wbkF* (C) with respect to their corresponding parental strains**. Mice were inoculated intraperitoneally with the doses (CFU/mouse) indicated in brackets for each strain. Significant differences: ** = *p* < 0.01, *** = *p* < 00.001.

The virulence and protection assessment in mice of Rev1::*wbdR*Δ*wbkC* and Rev 1 parental strain have been published previously [19]. For virulence assessment of H38Δ*wbkF* and Bov::CAΔ*wadB* mutants (this work), groups of 5 mice were inoculated intraperitoneally with the doses established as optimal (Figure 1) in preliminary experiments (not shown). The three vaccine candidates (Bov::CAΔ*wadB*, Rev1::*wbdR*Δ*wbkC* and H38Δ*wbkF*) were inoculated at 10^8^ CFU/mouse, a dose considered optimal for immunization with R mutants in previous experiments in mice [30, 32]; BoPA and Bov::CA parental strains were inoculated at 5 × 10^6^ CFU/mouse, as established previously [31]. Also, the lowest dose of *B. melitensis* H38 (10^4^ CFU/mouse) inducing a suitable spleen colonization was determined in preliminary experiments (not shown). Mice were euthanized at two different time points, selected for each mutant according previous works [19, 31, 32]: the first point corresponds to the expected “peak of multiplication” (week 1 and week 3 for *B. melitensis* and *B. ovis* strains, respectively) and the second one, to a later stage of infection (week 5 or 8). The residual virulence was determined by plating spleen homogenates on the appropriate agar medium (see above) and calculating the mean log_10_ CFU/spleen ± SD [41].

To evaluate the protective efficacy, groups of 5 mice were vaccinated subcutaneously with 10^8^ CFU of each vaccine candidate [19, 32]. Controls were similar groups vaccinated with 10^5^ CFU of Rev 1 or inoculated with BSS as a placebo vaccine. All mice were challenged with 5 × 10^6^ CFU of BoPA-Km^R^ (see above) administered intraperitoneally 4 weeks after vaccination. The mean log_10_ CFU/spleen ± SD of the challenge strain in each group was determined 2 weeks later by plating spleen homogenates on BAB-S supplemented with kanamycin (50 μg/mL) and incubation for 3–5 days in 10% CO_2_. Results were compared statistically (one-way ANOVA and Fisher’s Protected Least Significant Differences –PLSD- post-hoc tests) with those of unvaccinated (BSS) and Rev 1 vaccinated controls.

### Ram vaccination and challenge

The experiment in rams was performed in compliance with the current European (86/609/EEC) and Spanish (RD 53/2013) legislations on the use and protection of experimental animals.

Sixty-nine 4-month-old brucellosis-free Rasa Aragonesa rams of similar weight and body condition were randomly allotted and maintained in five separate pens throughout the experiment. To prepare the inocula, suspensions of bacteria (grown and harvested in BSS as described above) were adjusted spectrophotometrically (600 nm) to the appropriate concentration (10^9^ CFU/mL for Rev 1, 10^10^ CFU/mL for the mutant candidates and 10^11^ CFU/mL for *B. ovis* PA challenge strain). Purity, absence of S-R dissociation (when appropriate) and exact doses (triplicate measurements) were assessed retrospectively [40]. In the same day, three groups of 14 rams each were immunized subcutaneously in the left elbow with 4 mL of each vaccine candidate. As controls, a fourth group (*n* = 14) was vaccinated with 2 mL of Rev 1 and a fifth group (*n* = 13) was kept unvaccinated. Retrospective assessment showed that each ram received 9.6 × 10^9^ CFU of Bov::CAΔ*wadB*, 2.5 × 10^10^ CFU of Rev1::*wbdR*Δ*wbkC*, 2.5 × 10^10^ CFU of H38Δ*wbkF* and 1.6 × 10^9^ CFU of Rev 1. In the next two weeks, all rams were inspected for rectal temperature and local reactions at the inoculation site every two days.

Eight months after vaccination (when Rev 1 and the vaccine candidates had been cleared [33]), rams were challenged with 2.5 × 10^9^ CFU in 100 µL (determined by retrospective assessment) of *B. ovis* PA given by both conjunctival (50 µL) and preputial (50 µL) routes, and then clinically examined at weekly intervals. Eight weeks after challenge, they were euthanatized for bacteriological examination. For this, large portions of spleen and epididymides, the whole vesicular glands and ampullae, and the whole cranial (submaxillary, parotid and retropharyngeal), iliac, scrotal, prefemoral and superficial cervical lymph nodes were collected and submitted to culture on a suitable selective medium (see below). Briefly, each organ sample was degreased, superficially sterilized by gentle burning and homogenized in minimal amount of BSS (1 mL per 10 g of tissue, approx.) using a stomacher. Each tissue homogenate was cultured by plating 1 mL in each of duplicate plates of CITA medium [42]. Cultures were examined under a stereomicroscope after 5–7 days of incubation at 37 °C in a 10% CO_2_ atmosphere, and suspicious colonies streaked on BAB-S plates for identification by both standard procedures [40] and Bruce-ladder multiplex PCR [43]. A ram was classified as infected if at least one *B. ovis* PA CFU was isolated from any of the organs or lymph nodes sampled. The infection level of each sample was semi-quantitatively scored as follows: level 1, 1–5 CFU/plate; level 2, 6–25 CFU/plate; level 3, 26–125 CFU/plate; level 4, 126–250 CFU/plate; and level 5, > 250 CFU/plate (samples with level equal or higher than 3 were considered as severely infected, and rams showing at least one severely infected organ were counted as severely infected animals). Statistical comparisons of numbers of *B. ovis* infected (and severely infected) animals were made by Chi-square test (Fisher-Yates correction was applied when required). Numbers of infected (and severely infected) organs per animal were compared by STEPBOOT MULTTEST (5.0, SAS Institute Inc. Copyright©).

For serological studies, blood samples were taken before vaccination, one week after vaccination and every two or three weeks (including one week after challenge) until the end of the experiment.

### Serological tests

The Rose Bengal (RBT) and the complement fixation (CFT) tests for S *Brucella* were performed according to standard methods [34], and the S-LPS iELISA was performed as described before [44] using gold standard sera (from 46 *B. melitensis* culture positive and 78 brucellosis-free sheep) to determine the appropriate cut-off. The agar gel immunodiffusion (AGID) test for the serodiagnosis of *B. ovis* [45] was performed in 3 mm thick gels of 1% Noble Agar (Difco, USA) in borate buffer (0.1 M, pH 8.3)-10% NaCl using the recommended *B. ovis* REO 198 hot saline (HS) extract (rich in outer membrane proteins and R-LPS [46]) as antigen, and presence of precipitation bands was examined after 48 h of incubation at room temperature in a humid chamber. In addition, depending on the vaccine candidate (see “Results”), the antibody response was also examined by indirect ELISA (iELISA) using the appropriate antigen as described below.

The iELISA with HS extract of either *B. ovis* PA or Bov::CAΔ*wadB* was performed on standard 96-well polystyrene plates (Costar ®, USA). Optimal coating conditions (antigen at 2.5 µg/mL in carbonate buffer [0.06 M, pH 9.6] at 37 °C overnight), serum dilution (1/100) and conjugate (recombinant Protein G-HRPO, Thermo Fisher Scientific, USA at 0.2 μg/mL) were determined by previous titration with gold-standard sera from 46 *B. ovis* culture-positive and 78 brucellosis-free sheep. A 0.1% solution of 2, 2-azinobis, 3-ethyl-benzothiazoline sulfonic acid diammonium salt (ABTS; Sigma Chemical Co., USA) with 0.004% H_2_O_2_ in citrate buffer (0.05 M, pH 4) was used as the substrate, and optical density readings (OD_405nm,_ Labsystems Multiskan RC) obtained after 15 min of incubation at room temperature. Results were expressed as the percentage of OD_405nm_ with respect to a positive control serum. Under these conditions, sera showing % OD_405nm_ above 40% (cut-off resulting in 100% specificity and maximum [98%] sensitivity with the gold standard sera) were considered positive.

An iELISA with N-acetyl-perosamine O-PS was performed using a LPS rich extract of the previously described *B. abortus* 2308 W *wbdR*Δ*wbkC* construct [19]. This *wbdR*Δ*wbkC-*iELISA was optimized using the same panel of gold-standard sera employed in the standard S-LPS ELISA (see above). Costar ® plates were coated with 2.5 μg/mL of antigen in carbonate buffer (pH 9.6) at 37 °C overnight. Sera were tested at 1/100 dilution and the Protein-G-HRP (0.2 μg/mL) and the ABTS-H_2_O_2_ mixture described above were used as conjugate and substrate, respectively. The OD_405nm_ readings and % OD_405nm_ were obtained as described above and sera showing %OD_405nm_ ≥ 50% (optimal cut-off) were scored as positive.

## Results

### Characterization of vaccine candidates

Table 1 summarizes the relevant characteristics of the vaccine candidates investigated. Analyses by SDS-PAGE and Western blot with monoclonal antibody A68/24G12/A08 (which discriminates intact and truncated *Brucella* LPS core oligosaccharides [36]) and polyclonal sera of the appropriate specificity [39], confirmed that the three candidates displayed the phenotype predicted according to previous works with homologous or heterologous *Brucella* mutants and constructs: (i) H38Δ*wbkF* carried a R-LPS with a complete core; (ii) Bov::CAΔ*wadB* R-LPS showed the molecular weight shift and epitope defect that correspond to a truncated LPS core lateral branch; and (iii) Rev1::Tn7*wbdR*Δ*wbkC* displayed the shorter O-PS and the epitopic changes associated with the presence of N-acetyl-perosamine and absence of N-formyl-perosamine in the O-PS (Additional file 3).

**Table 1:** **Vaccine candidates**.

*Vaccine*	Genetic characteristics	Relevant phenotype	References
Rev1::*wbdR*Δ*wbkC*	Rev 1 *wbkC* (formyl-transferase gene) deleted mutant carrying a mini-Tn7 insert with *E. coli* O157:H7 *wbdR* (acetyl-transferase)	S-LPS with N-acetyl-perosamine O-PS	[19]
H38Δ*wbkF*	*B. melitensis* H38 *wbkF* (bactoprenol priming for O-PS polymerization gene) deleted mutant	R-LPS with intact core oligosaccharide	This work
Bov::CAΔ*wadB*	*B. ovis* PA *wadB* (core sugar glycosyl transferase gene) deleted mutant carrying a mini-Tn7 insert with the carbonic anhydrases (CAI and CAII) genes of *B. abortus* 2308 W	CO_2_-independent; R-LPS with a truncated core oligosaccharide	This work. Δ*wadB* and::CA single mutants in [27] and [30], respectively

### Residual virulence and protection in mice

These studies showed that the three vaccine candidates have attenuated virulence profiles with respect to their virulent counterparts (Figure 1). Bov::CAΔ*wadB* (Figure 1A) showed significantly lower CFU/spleen numbers than *B. ovis* PA virulent strains at weeks 3 (expected peak of multiplication for *B. ovis*) and 8 after inoculation. Attenuation with respect to the parental strain was clearly observed also for Rev1::*wbdR*Δ*wbkC* (Figure 1B, from [19]) at post-inoculation weeks 1 (peak of multiplication for *B. melitensis*) and 5. H38Δ*wbkF* (Figure 1C) yielded similar CFU/spleen numbers to the parental strain at week 1, but the mutant attenuation was evident at later stages (week 5).

Using this model, we tested all vaccine candidates for protection against *B. ovis* in two successive experiments. As can be seen in Table 2, the results obtained with all vaccine candidates were not statistically different from those obtained with the Rev 1 vaccine.

**Table 2:** **Protection against *B. ovis* PA-Km**^**R**^** in mice**.

Experiment	Vaccine (CFU dose)	*B. ovis* PA-Km^R^ (mean ± SD of log_10_ CFU/spleen)	Units of protection^a^
1	Bov::CAΔ*wadB* (10^8^)	2.46 ± 1.68^b,c^	3.6
	H38Δ*wbkF* (10^8^)	3.42 ± 0.62^b,c^	2.6
	Rev 1 (10^5^)	2.50 ± 1.49^b^	3.5
	Placebo (BSS)	6.04 ± 0.46	0
2	Rev1::*wbdR*Δ*wbkC* (10^8^)	2.16 ± 0.95^b,c^	4.1
	Rev 1 (10^5^)	2.45 ± 1.35^b^	3.8
	Placebo (BSS)	6.26 ± 0.16	0

^a^Units of protection: average log_10_ CFU of challenge strain in the spleens of placebo (BSS) controls minus average of log_10_ CFU of the challenge strain in the spleens of vaccinated mice. ^b^Significant differences (*P* < 0.01) versus the placebo (BSS) control group (ANOVA and Fisher’s PSLD test); ^c^No significant differences versus the Rev 1 vaccinated group (ANOVA and Fisher’s PSLD test).

### Protection in rams

No relevant clinical signs were evidenced after vaccination. The mean rectal temperature remained normal in the animals inoculated with the three vaccine candidates. Some Rev 1 vaccinated rams showed a slight increase in body temperature and an inflammatory reaction at the inoculation site that was resolved in a few weeks. Upon challenge, no vaccinated ram developed clinical testicular lesions but these were observed in two unvaccinated rams.

**Figure 2:**
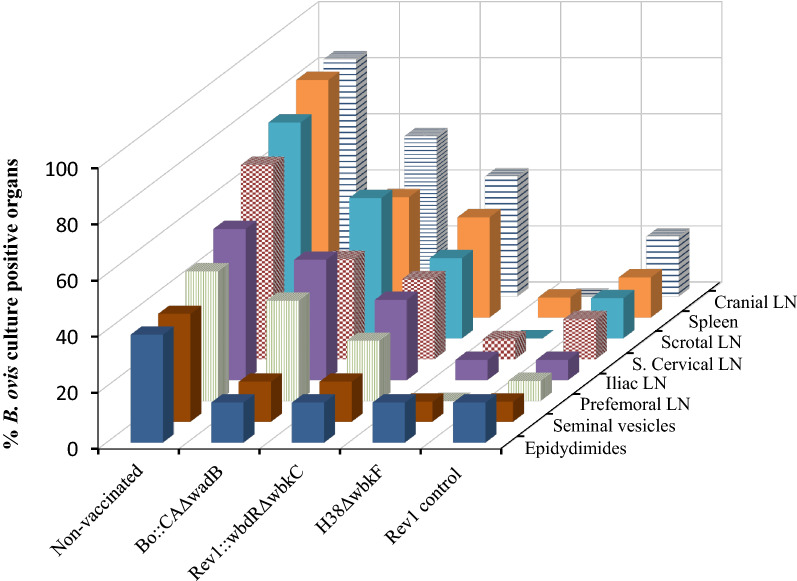
**Percentages of *B. ovis* culture positive organs in each experimental group of vaccinated rams**.

The results of the thorough bacteriological search after necropsy are summarized in Table 3 and Figure 2. Taken together, the results of control groups show that the experimental conditions resulted in values suitable for statistical comparisons (84.6% vs. 21.4% infected rams in the unvaccinated and Rev 1 vaccinated groups, respectively; *P* < 0.05). The protection conferred by Bov::CAΔ*wadB* was manifestly not significant (78.6% of infections) and that of the Rev1::*wbdR*Δ*wbkC* candidate (50% of infections) not statistically different from that of the unvaccinated control group. On the other hand, H38Δ*wbkF* conferred a level of protection (16.7% of infections) similar to that obtained with Rev 1 (Table 3), with slightly lower percentages of infected organs and no isolation from cranial, scrotal or prefemoral lymph nodes (Figure 2). These results were paralleled in the numbers of infected organs (Table 3) (5.4% and 10.7% for H38Δ*wbkF* and Rev 1, respectively) and of severely infected animals/organs (7.1%/1.8% and 7.1%/0.9% for H38Δ*wbkF* and Rev 1, respectively).

**Table 3:** **Protective efficacy of vaccine candidates against a *B. ovis* PA experimental challenge in rams**.

Vaccine (CFU dose)	No. infected animals/total (%)^a^	No. infected organs/total (%)^b^	No. severely infected animals (%)^a,c^/no. severely infected organs (%)^b^
Bov::CAΔ*wadB* (1 × 10^10^)	11/14 (78.6)	41/112 (36.6)	4 (28.6)^d^/11 (9.8)^d^
Rev1::*wbdR*Δ*wbkC* (2.5 × 10^10^)	7/14 (50.0)	30/112 (26.8)^d^	5 (35.7)/13 (11.6)^d^
H38Δ*wbkF* (2.5 × 10^10^)	2/14 (16.7)^e,f^	6/112 (5.4)^e,f^	1 (7.1)^e,f^/2 (1.8)^e,f^
Rev 1 (2 × 10^9^)	3/14 (21.4)^d^	12/112 (10.7)^e^	1 (7.1)^e^/1 (0.9)^e^
Unvaccinated	11/13 (84.6)	64/104 (61.5)	9 (69.2)/30 (28.8)

^a^Statistical comparisons by Chi-square test (with Fisher-Yates correction when required). ^b^Statistical comparisons by STEPBOOT MULTTEST (SAS); ^c^severely infected animals were those showing at least one organ sample with an infection level ≥ 3 (≥ 26 CFU/plate). ^d^Significant difference (*P* < 0.05) versus unvaccinated control; ^e^High significant difference (*P* < 0.001) vs. unvaccinated control; ^f^no significant (*P* > 0.05) versus Rev 1 vaccinated group.

### Serological responses in rams

**Figure 3:**
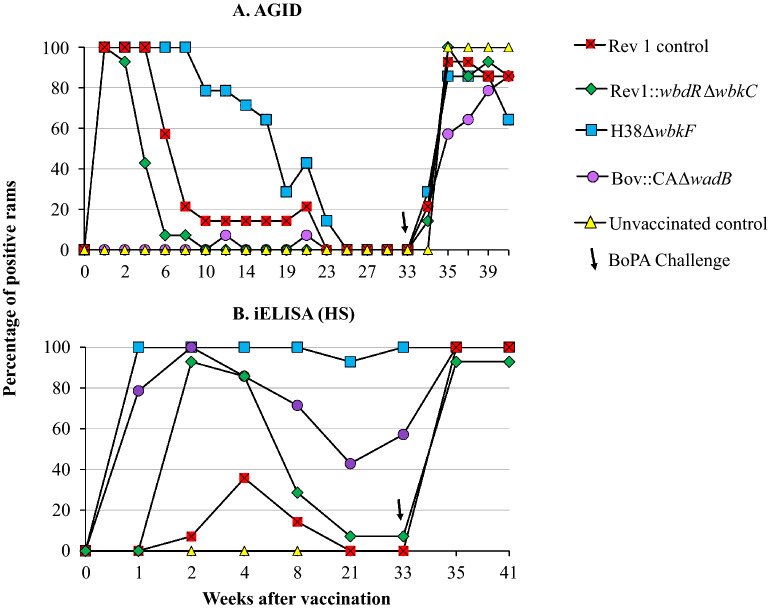
**Evolution of the percentages of positive rams after vaccination (week 0) and challenge (week 33) in: (A) AGID; and (B) iELISA using *B. ovis* HS extracts**.

Figure 3 presents the results of the antibody response to the *B. ovis* HS envelope antigens generated in vaccinated rams before and after challenge. All rams were positive in the AGID assay by week 1 after immunization, with the notable exception of those vaccinated with Bov::CAΔ*wadB* among which very few positive responses were detected before challenge (Figure 3A). In the Rev1::*wbdR*Δ*wbkC* and Rev 1 vaccinated groups, AGID-positive responses decreased rapidly and all animals became negative by weeks 10 and 23, respectively (Figure 3A). As judged by the percentage of AGID-positive animals, H38Δ*wbkF* elicited the most durable antibody response, with over 60% of positive animals until week 17 (Figure 3A). No matter the experimental group, all rams were AGID-negative before challenge (week 33). When assessed by iELISA (Figure 3B), the percentage of positive animals in the groups vaccinated with Bov::CAΔ*wadB* and H38Δ*wbkF* were higher and more persistent than those observed in the AGID test and only the group vaccinated with Rev1::*wbdR*Δ*wbkC* was totally negative by the time of challenge. Interestingly, when sera from Bov::CAΔ*wadB* vaccinated rams were studied in the iELISA with a homologous HS antigen, 100% of them were positive from week 1 until the end of the experiment (not shown). Upon challenge, most animals became both AGID and HS iELISA-positive, as expected.

**Figure 4:**
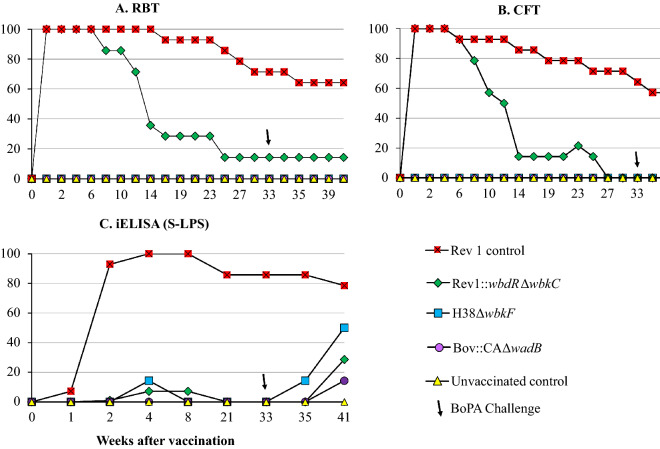
**Evolution of the percentages of positive rams in S-LPS tests after vaccination (week 0) and challenge (week 33): (A) RBT; (B), CFT; and (C) iELISA**.

Figure 4 presents the results obtained with serodiagnostic tests for S brucellae. Unsurprisingly, all animals vaccinated with Rev 1 were positive in RBT, CFT and S-LPS iELISA by week 2 and a high percentage remained positive thereafter. Although Rev1::*wbdR*Δ*wbkC* bears a modified O-PS, all rams vaccinated with this candidate were positive in both RBT and CFT during the first weeks but the reactions persisted less than those induced by Rev 1. However, only very few and transient reactions were detected in the S-LPS iELISA in these Rev1::*wbdR*Δ*wbkC* vaccinated rams (Figure 4C), even though these rams showed a more intense and persistently positive response in the *wbdR*Δ*wbkC* iELISA (Additional file 4). In contrast with Rev 1 and Rev1::*wbdR*Δ*wbkC* vaccinates, no ram immunized with H38Δ*wbkF* or Bov::CAΔ*wadB* showed antibodies detectable in either RBT or CFT, and only a few of those vaccinated with H38Δ*wbkF* showed a transiently positive and relatively early response in the S-LPS iELISA before challenge (Figure 4). As expected, the proportion of RBT and CFT positive animals did not increase after the challenge for any of the experimental groups (Figures 4A and B) but 50% and less than 20%, respectively, of H38Δ*wbkF* and Bov::CAΔ*wadB* vaccinated rams reacted then in the S-LPS iELISA (Figure 4C).

## Discussion

As underlined in the Introduction, there is a need for a *B. ovis* vaccine to replace Rev 1, banned in areas where *B. melitensis* has been eradicated or has never been present. Following the evidence that live-attenuated vaccines are advantageous for triggering protective responses against *Brucella* [47], in this work we investigated three vaccine candidates of this kind.

It is known that *Brucella* behaves as a stealthy parasite that avoids detection by innate immunity at the onset of infection, thus retarding the adaptive response and facilitating to reach intracellular niches [48]. This furtive behavior is related to the absence of marked pathogen-associated molecular patterns in envelope molecules, chiefly in the LPS [48]. Immunological, genetic and structural investigations have established that the core of *Brucella* LPS carries a pentasaccharide branch that hinders recognition of the lipid A-inner core by the TLR4-MD2 receptor system of innate immunity [27, 36, 49, 50]. Hence, mutations preventing the assembly of this pentasaccharide bolster Th1 immunoresponses, thereby causing attenuation [27, 36, 49, 50]. Consequently, disruption of the core lateral branch was proposed as a strategy to develop brucellosis vaccines [51] and previous work with *B. ovis* shows that the core glycosyltransferase gene *wadB* is an appropriate target for this purpose [27]. Yet, we found that, when combined with CA genes of *B. abortus* to overcome the practical limitations of CO_2_-dependence, the *B. ovis wadB* mutant was totally ineffective as vaccine in rams even though it was protective in mice. These results mean that the mouse model failed utterly to predict a positive vaccine result in the natural host, and are in line with other data that strongly suggest that its usefulness in the search for a brucellosis live vaccine is limited to screen out overattenuated or none protective candidates [32, 33, 52, 53].

Nevertheless, the total failure of Bov::CAΔ*wadB* as a vaccine was unexpected and, although the explanation is far from obvious, it is worthwhile to speculate on the reasons. Noteworthy, proportions of Bov::CAΔ*wadB* vaccinated rams positive in AGID and iELISA with *B. ovis* HS were respectively lower than those in any other vaccinated group. Since all rams remained positive in the iELISA with HS of the homologous Bov::CAΔ*wadB*, and HS is rich in R-LPS [46], these results evidence the importance of the epitopes of the core branch in the antibody response to *B. ovis* LPS. In mice, a role for antibodies in *B. ovis* protective immunity has been shown in passive transfer assays with monoclonal antibodies to envelope antigens and with sera from mice immunized with RB51 (reviewed in [35]). However, vaccination with HS in sheep does not show a correlation between protection and the intensity of the antibody response to these antigens [20, 35]. On the other hand, experiments with the same antigens in several adjuvant formulations provide clear evidence that a strong Th1 cell-mediated immunoresponse is important in protection in sheep [21, 35].

Therefore, although the lack of antibodies to the full core may play a role, we favor the hypothesis that the failure of Bov::CAΔ*wadB* to protect against *B. ovis* was caused by an inappropriate Th1 response. This may seem paradoxical because the LPS core defect has been shown to increase recognition by LPS receptors (see above), thereby bolstering pro-inflammatory cytokine production and initiating the Th1 response at earlier times than the wild- type [49]. However, this comparatively early Th1 response accelerates clearance of *Brucella* core mutants in mice [49] and, as discussed below, a given permanence in the host is necessary for a vaccine to generate protective immunity. Thus, it could be that Bov::CAΔ*wadB* clearance was too rapid to generate such a response. Investigation of these hypotheses could help to clarify the virulence mechanisms of *B. ovis* and the immunity to this comparatively less studied species, knowledge with which it may be possible to develop an homologous *B. ovis* vaccine.

We also examined the Rev1::*wbdR*Δ*wbkC* construct. Based on the results in mice [19], the premises were that this candidate could protect sheep against *B. ovis* and elicit antibodies that, while not interfering in standard S-LPS tests, could be useful to identify vaccinated animals. Concerning protection, we found that it was insufficient, even though in this case the differences with the results in mice were not as dramatic as those of Bov::CAΔ*wadB*. It is worth pointing out that since the first studies with vaccine RB51 a much larger dose has been found necessary to show that some R mutants match the protection obtained with S19 or Rev 1 (reference OIE S vaccines) in mice [16, 32, 52, 53], and this is also true for Rev1::*wbdR*Δ*wbkC* (Table 2 and [19]). However, to reproduce in the natural host the 3 log dose increase applied in the mouse model is not feasible (doses over 10^10^ CFU are not practical in sheep). Therefore, we used a dose equivalent to that of R vaccines (1–5 × 10^10^, [33]). Thus, the contrasting results obtained with Rev1::*wbdR*Δ*wbkC* in rams and in mice are likely to reflect the laboratory bias in dose introduced for screening purposes and the limitations of the mouse model.

Why Rev1::*wbdRΔwbkC* being smooth could require a dose larger than Rev 1 in the mouse model may reflect a defect in cell entry and/or control of intracellular trafficking caused by the O-PS modification because both are critical processes in the biology of S *Brucella* in which the wild-type O-PS plays an important role [54, 55]. Concerning the specificity of the antibodies, our results show that the change in O-PS epitopic structure of Rev1::*wbdR*Δ*wbkC* is insufficient to fully abrogate the interference in standard S-LPS tests (Figure 4) or to differentiate vaccinated and infected rams (Additional file 4). These results, which are reminiscent of the reactivity of IgG of infected cattle with the O-PS of the *E. hermanii* serotypes that carry N-acetyl-perosamine [56] set another difference with the mouse model [19] and discourage a practical use of this vaccine epitopic tagging.

In contrast with the other candidates, H38Δ*wbkF* generated protection in rams similar to that obtained with reference Rev 1 vaccine. In previous works, we found that an intact LPS core is necessary for an R vaccine to provide maximal protection in at least the mouse model, and *wbkF* mutants meet this requisite [16, 32]. Also in keeping with the predicted function of WbkF (Table 1), they are unable to synthesize internal O-PS precursors that would elicit anti-O-PS antibodies, an undesirable effect in an *R* vaccine that occurs in *wadA* (formerly wa**) and *wzm* R mutants [32, 33, 57, 58]. Moreover, as indicated in the Introduction, this candidate was not selected only by the results in mice. In a previous work [33], we found that a *B. melitensis* H38*wbkF*::Tn5 Km^R^ mutant had an organ distribution and persistence in sheep like those of Rev 1, strongly suggesting similarly intense antigenic stimuli. All these characteristics made strain H38 and gene *wbkF* attractive for developing a *B. ovis* vaccine, and for a potential use we constructed the mutant by deletion (to avoid any potential reversibility) without antibiotic-resistance markers. Thus, although we did not evaluate again the kinetics of infection of the H38Δ*wbkF* mutant in rams, its almost total identity with the H38*wbkF*::Tn5 Km^R^ should result in a distribution and persistence similar to that of Rev 1 and, in fact, both elicited similarly persistent positive responses in the *B. ovis* HS-AGID.

As predicted, vaccination with H38Δ*wbkF* did not generate a serological interference in either RBT or CFT, tests that detect anti-O-PS antibodies [3]. However, transient reactions were observed in the S-LPS ELISA, and the interference in this assay (but not in the RBT and CFT) was evident after challenge. This was not unexpected since R vaccines and *B. ovis* induce antibodies to the core epitopes shared by the R and S-LPS that, while not accessible in tests like RBT and CFT, become exposed to antibodies in ELISA and in the fluorescence polarization assay [3]. Therefore, if H38Δ*wbkF* (or any other R vaccine) is employed in *B. melitensis-*free areas where the latter two tests are used for surveillance, this cross-reactivity should be considered and the simple and inexpensive RBT implemented as a parallel test in doubtful cases. A more difficult practical problem is posed by the interference of H38Δ*wbkF* in *B. ovis* serodiagnostic tests. Although more persistent in iELISA than in AGID, in which all animals were negative 27 weeks after vaccination (Figure 3), this is a protracted response that may hamper implementation of test and slaughter combined with H38Δ*wbkF* vaccination for the eradication of *B. ovis* infection. More information is necessary to better assess the effects of route of vaccination, vaccine dose and the optimal age of sheep at vaccination on this diagnostic interference, as well as the efficacy of the H38Δ*wbkF* vaccine under field conditions and the potential effects that anamnestic responses after contacts with *B. ovis* field strains may have in vaccinated animals. Given the importance of ewes in the epidemiology of *B. ovis* [10], the vaccination of female sheep would be essential to a suitable control of infection. Like Rev 1, at least the *B. melitensis* B115 spontaneous *wzm* R mutant is highly abortifacient in pregnant sheep [58] and this and other potential untoward effects of the H38Δ*wbkF* vaccine in adult sheep need to be investigated. Research is in progress to further define the conditions of field use of H38Δ*wbkF*.

## Supplementary Information

**Additional file 1:** **Primers used for mutant construction**.

**Additional file 2:** ***E. coli*** **S17 λpir and plasmids used for mutant construction**.

**Additional file 3:** **LPS characterization of vaccine candidates by SDS-PAGE electrophoresis-silver staining and Western blot analyses of SDS-proteinase K extracts.** Upper panels (from [19]): Analyses of (1) Rev 1, (2) Rev1::Tn7*wbdR*, and (3) Rev1::Tn7*wbdR*Δ*wbkC* extracts. Lower panels: Analyses of (4) H38, (5) H38Δ*wbkF*, (6) Bov::CA and (7) Bov::CAΔ*wadB* extracts.

**Additional file 4:** **Reactivity (%OD) in iELISA using N-formyl-perosamine wild-type S-LPS (A) or N-acetyl-perosamine S-LPS from a** *wbdR***Δ*wbkC*** **construct (B) and 46 sera from** ***B. melitensis*** **culture positive (C+) sheep, 78 brucellosis-free (BF) sheep and rams immunized with Rev1::*wbdR*Δ*wbkC***.

## References

[1] McDermott J, Grace D, Zinsstag J (2013). Economics of brucellosis impact and control in low-income countries. Rev Sci Tech.

[2] Moreno E (2020). The one hundred year journey of the genus *Brucella* (Meyer and Shaw 1920). FEMS Microbiol Rev.

[3] Ducrotoy MJ, Conde-Álvarez R, Blasco JM, Moriyón I (2016). A review of the basis of the immunological diagnosis of ruminant brucellosis. Vet Immunol Immunopathol.

[4] Zygmunt MS, Blasco JM, Letesson JJ, Cloeckaert A, Moriyón I (2009). DNA polymorphism analysis of *Brucella* lipopolysaccharide genes reveals marked differences in O-polysaccharide biosynthetic genes between smooth and rough *Brucella* species and novel species-specific markers. BMC Microbiol.

[5] Tsolis RM, Seshadri R, Santos RL, Sangari FJ, Lobo JM, de Jong MF, Ren Q, Myers G, Brinkac LM, Nelson WC, Deboy RT, Angiuoli S, Khouri H, Dimitrov G, Robinson JR, Mulligan S, Walker RL, Elzer PE, Hassan KA, Paulsen IT (2009). Genome degradation in *Brucella ovis* corresponds with narrowing of its host range and tissue tropism. PLoS One.

[6] Picard-Hagen N, Berthelot X, Champion JL, Eon L, Lyazrhi F, Marois M, Peglion M, Schuster A, Trouche C, Garin-Bastuji B (2015). Contagious epididymitis due to *Brucella ovis*: relationship between sexual function, serology and bacterial shedding in semen. BMC Vet Res.

[7] ECDC (2019) European Centre for Disease Prevention and Control. Brucellosis. In: ECDC. Annual epidemiological report for 2017. Stockholm, Sweden. https://www.ecdc.europa.eu/sites/default/files/documents/brucellosis-annual-epidemiological-report-2017.pdf.

[8] Moreno E (2014). Retrospective and prospective perspectives on zoonotic brucellosis. Front Microbiol.

[9] Marco J, González L, Cuervo LA, De Heredia FB, Barberán M, Marín C, Blasco JM (1994). *Brucella ovis* infection in two flocks of sheep. Vet Rec.

[10] Blasco JM, Lefèvre PC, Blancou JC, Chermette R, Uilenberg G (2010). *Brucella Ovis* Infection. Infectious and parasitic diseases of livestock.

[11] Ridler AL, West DM (2011). Control of *Brucella ovis* infection in sheep. Vet Clin North Am Food Anim Pract.

[12] Blasco JM, Neilsen K, Duncan JR (1990). Brucella ovis. Animal Brucellosis.

[13] Marín CM, Mainar RC, de Miguel MJ, Andrés-Barranco S, Álvarez JJ, Blasco JM, Muñoz PM (2019) Re-emergence of *Brucella ovis* infection in Aragón (Spain) after the ban of Rev 1 vaccination In: International Brucellosis Society Meeting, Chicago, EEUU. https://citarea.cita-aragon.es/citarea/bitstream/10532/4864/1/2019_420ab.pdf

[14] El Idrissi AH, Benkirane A, El Maadoudi M, Bouslikhane M, Berrada J, Zerouali A (2001). Comparison of the efficacy of *Brucella abortus* strain RB51 and *Brucella melitensis* Rev 1 live vaccines against experimental infection with *Brucella melitensis* in pregnant ewes. Rev Sci Tech.

[15] Jiménez de Bagüés MP, Barberán M, Marín CM, Blasco JM (1995). The *Brucella abortus* RB51 vaccine does not confer protection against *Brucella ovis* in rams. Vaccine.

[16] Monreal D, Grilló MJ, Gonzalez D, Marín CM, De Miguel MJ, Lopez-Goñi I, Blasco JM, Cloeckaert A, Moriyon I (2003). Characterization of *Brucella abortus* O-polysaccharide and core lipopolysaccharide mutants and demonstration that a complete core is required for rough vaccines to be efficient against *Brucella abortus* and *Brucella ovis* in the mouse model. Infect Immun.

[17] Grilló MJ, Marín CM, Barberan M, de Miguel MJ, Laroucau K, Jacques I, Blasco JM (2009). Efficacy of bp26 and bp26/omp31 *B. melitensis* Rev. 1 deletion mutants against* Brucella ovi*s in rams. Vaccine.

[18] Zabalza-Barangua A, San Román B, Chacón-Díaz C, de Miguel MJ, Munoz PM, Iriarte M, Blasco JM, Grilló MJ (2018). GFP tagging of *Brucella melitensis* Rev1 allows the identification of vaccinated sheep. Transbound Emerg Dis.

[19] Aragón-Aranda B, de Miguel MJ, Martínez-Gómez E, Zúñiga-Ripa A, Salvador-Bescós M, Moriyón I, Iriarte M, Muñoz PM, Conde-Álvarez R (2019). Rev 1 *wbdR* tagged vaccines against *Brucella ovis*. Vet Res.

[20] Blasco JM, Gamazo C, Winter AJ, Jiménez de Bagüés MP, Marín C, Barberán M, Moriyón I, Alonso-Urmeneta B, Díaz R (1993). Evaluation of whole cell and subcellular vaccines against *Brucella ovis* in rams. Vet Immunol Immunopathol.

[21] Da Costa MR, Irache JM, Blasco JM, Muñoz MP, Marín CM, Grilló MJ, De Miguel MJ, Barberán M, Gamazo C (2010). Evaluation of particulate acellular vaccines against *Brucella ovis* infection in rams. Vaccine.

[22] Díaz AG, Quinteros DA, Paolicchi FA, Rivero MA, Palma SD, Pardo RP, Clausse M, Zylberman V, Goldbaum FA, Estein SM (2019). Mucosal immunization with polymeric antigen BLSOmp31 using alternative delivery systems against *Brucella ovis* in rams. Vet Immunol Immunopathol.

[23] Estein SM, Fiorentino MA, Paolicchi FA, Clausse M, Manazza J, Cassataro J, Giambartolomei GH, Coria LM, Zylberman V, Fossati CA, Kjeken R, Goldbaum FA (2009). The polymeric antigen BLSOmp31 confers protection against *Brucella ovis* infection in rams. Vaccine.

[24] Silva AP, Macedo AA, Costa LF, Rocha CE, García LN, Farias JR, Gomes PP, Teixeira GC, Fonseca KW, Maia AR, Neves GG, Romao EL, Silva TM, Mol JP, Oliveira RM, Araujo MS, Nascimento EF, Martins-Filho OA, Brandao HM, Paixao TA, Santos RL (2015). Encapsulated *Brucella ovis* lacking a putative ATP-binding cassette transporter (DeltaabcBA) protects against wild type *Brucella ovis* in Rams. PLoS One.

[25] Silva AP, Macedo AA, Silva TM, Ximenes LC, Brandao HM, Paixao TA, Santos RL (2015). Protection of an encapsulated live attenuated strain of *Brucella ovis* (DeltaabcBA) against experimental challenge in the murine model. Clin Vaccine Immunol.

[26] Grilló MJ, Marín CM, Barberán M, Blasco JM (1999). Experimental *Brucella ovis* infection in pregnant ewes. Vet Rec.

[27] Soler-LLorens P, Gil-Ramírez Y, Zabalza-Barangua A, Iriarte M, Conde R, Zúñiga-Ripa A, San Román B, Zygmunt M, Vizcaíno N, Cloeckaert A, Grilló M-J, Moriyón I, Lopez-Goñi I (2014). Mutants in the lipopolysaccharide of *Brucella ovis* are attenuated and protect against *B. ovis* infection in mice. Vet Res.

[28] Sancho P, Tejedor C, Sidhu-Muñoz RS, Fernández-Lago L, Vizcaíno N (2014). Evaluation in mice of *Brucella**ovis* attenuated mutants for use as live vaccines against *B.**ovis* infection. Vet Res.

[29] Sidhu-Muñoz RS, Sancho P, Cloeckaert A, Zygmunt MS, de Miguel MJ, Tejedor C, Vizcaíno N (2018). Characterization of cell envelope multiple mutants of *Brucella ovis* and assessment in mice of their vaccine potential. Front Microbiol.

[30] Vizcaíno N, Pérez-Etayo L, Conde-Álvarez R, Iriarte M, Moriyón I, Zúñiga-Ripa A (2020). Disruption of pyruvate phosphate dikinase in *Brucella ovis* PA CO_2_-dependent and independent strains generates attenuation in the mouse model. Vet Res.

[31] Pérez-Etayo L, de Miguel MJ, Conde-Álvarez R, Muñoz PM, Khames M, Iriarte M, Moriyón I, Zúñiga-Ripa A (2018). The CO_2_-dependence of *Brucella ovis* and *Brucella abortus* biovars is caused by defective carbonic anhydrases. Vet Res.

[32] González D, Grilló MJ, De Miguel MJ, Ali T, Arce-Gorvel V, Delrue RM, Conde-Álvarez R, Muñoz P, López-Goñi I, Iriarte M, Marín CM, Weintraub A, Widmalm G, Zygmunt M, Letesson JJ, Gorvel JP, Blasco JM, Moriyón I (2008). Brucellosis vaccines: assessment of *Brucella melitensis* lipopolysaccharide rough mutants defective in core and O-polysaccharide synthesis and export. PLoS One.

[33] Barrio MB, Grilló MJ, Muñoz PM, Jacques I, González D, de Miguel MJ, Marín CM, Barberán M, Letesson JJ, Gorvel JP, Moriyón I, Blasco JM, Zygmunt MS (2009). Rough mutants defective in core and O-polysaccharide synthesis and export induce antibodies reacting in an indirect ELISA with smooth lipopolysaccharide and are less effective than Rev 1 vaccine against *Brucella melitensis* infection of sheep. Vaccine.

[34] OIE (2018) Brucellosis (*Brucella abortus, B. melitensis* and *B. suis*) (infection with *B. abortus, B. melitensis* and *B. suis*). NB: Version adopted in May 2016. In: Manual of Diagnostic Tests and Vaccines for Terrestrial Animals 2018 Off Int Epizoot, París, pp 1–44. https://www.oie.int/fileadmin/Home/fr/Health_standards/tahm/3.01.04_BRUCELLOSIS.pdf

[35] Muñoz PM, Estevan M, Marín CM, De Miguel MJ, Grilló MJ, Barberan M, Irache JM, Blasco JM, Gamazo C (2006). *Brucella* outer membrane complex-loaded microparticles as a vaccine against *Brucella ovis* in rams. Vaccine.

[36] Gil-Ramírez Y, Conde-Álvarez R, Palacios-Chaves L, Zúñiga-Ripa A, Grilló MJ, Arce-Gorvel V, Hanniffy S, Moriyón I, Iriarte M (2014). The identification of *wadB*, a new glycosyltransferase gene, confirms the branched structure and the role in virulence of the lipopolysaccharide core of *Brucella abortus*. Microb Pathog.

[37] Simon R, Priefer U, Pühler A (1983). A broad host range mobilization system for *in vivo* genetic engineering: transposon mutagenesis in Gram negative bacteria. Nat Biotechnol.

[38] Aragón-Aranda B, de Miguel MJ, Lázaro-Antón L, Salvador-Bescós M, Zúñiga-Ripa A, Moriyón I, Iriarte M, Muñoz PM, Conde-Álvarez R (2020). Development of attenuated live vaccine candidates against swine brucellosis in a non-zoonotic *B.**suis* biovar 2 background. Vet Res.

[39] Martínez-Gómez E, Stahle J, Gil-Ramírez Y, Zúñiga-Ripa A, Zaccheus M, Moriyón I, Iriarte M, Widmalm G, Conde-Álvarez R (2018). Genomic insertion of a heterologous acetyltransferase generates a new lipopolysaccharide antigenic structure in *Brucella abortus* and *Brucella melitensis*. Front Microbiol.

[40] Alton GG, Jones LM, Angus RD, Verger JM (1988) Techniques for the brucellosis laboratory. Institut National de la Reserche Agronomique, Paris, France. ISBN 2738000428

[41] Grilló MJ, Manterola L, de Miguel MJ, Muñoz PM, Blasco JM, Moriyón I, López-Goñi I (2006). Increases of efficacy as vaccine against *Brucella abortus* infection in mice by simultaneous inoculation with avirulent smooth *bvrS/bvrR* and rough *wbkA* mutants. Vaccine.

[42] De Miguel MJ, Marín CM, Muñoz PM, Dieste L, Grilló MJ, Blasco JM (2011). Development of a selective culture medium for primary isolation of the main *Brucella* Species. J Clin Microbiol.

[43] Lopez-Goñi I, García-Yoldi D, Marín CM, de Miguel MJ, Muñoz PM, Blasco JM, Jacques I, Grayon M, Cloeckaert A, Ferreira AC, Cardoso R, de Sa MIC, Walravens K, Albert D, Garin-Bastuji B (2008). Evaluation of a multiplex PCR assay (Bruce-ladder) for molecular typing of all *Brucella* species, including the vaccine strains. J Clin Microbiol.

[44] Muñoz PM, Boadella M, Arnal M, de Miguel MJ, Revilla M, Martínez D, Vicente J, Acevedo P, Oleaga A, Ruiz-Fons F, Marín CM, Prieto JM, de la Fuente J, Barral M, Barberán M, de Luco DF, Blasco JM, Gortazar C (2010). Spatial distribution and risk factors of Brucellosis in Iberian wild ungulates. BMC Infect Dis.

[45] OIE (2018) Ovine epidydimitis (*Brucella ovis*). Chapter 3.7.7. In: Manual of Diagnostic Tests and Vaccines for Terrestrial Animals (Version adopted by the world assembly of OIE delegates in May 2018). Paris, France. https://www.oie.int/fileadmin/Home/fr/Health_standards/tahm/3.07.07_OVINE_EPID.pdf

[46] Riezu-Boj JI, Moriyón I, Blasco JM, Gamazo C, Díaz R (1990). Antibody response to *Brucella ovis* outer membrane proteins in ovine brucellosis. Infect Immun.

[47] Pandey A, Cabello A, Akoolo L, Rice-Ficht A, Arenas-Gamboa A, McMurray D, Ficht TA, de Figueiredo P (2016). The case for live attenuated vaccines against the neglected zoonotic diseases Brucellosis and Bovine Tuberculosis. PLoS Negl Trop Dis.

[48] Barquero-Calvo E, Chaves-Olarte E, Weiss DS, Guzmán-Verri C, Chacón-Díaz C, Rucavado A, Moriyón I, Moreno E (2007). *Brucella abortus* uses a stealthy strategy to avoid activation of the innate immune system during the onset of infection. PLoS One.

[49] Conde-Alvarez R, Arce-Gorvel V, Iriarte M, Mancek-Keber M, Barquero-Calvo E, Palacios-Chaves L, Chacon-Diaz C, Chaves-Olarte E, Martirosyan A, von Bargen K, Grillo MJ, Jerala R, Brandenburg K, Llobet E, Bengoechea JA, Moreno E, Moriyon I, Gorvel JP (2012). The lipopolysaccharide core of *Brucella abortus* acts as a shield against innate immunity recognition. PLoS pathog.

[50] Fontana C, Conde-Álvarez R, Stahle J, Holst O, Iriarte M, Zhao Y, Arce-Gorvel V, Hanniffy S, Gorvel JP, Moriyón I, Widmalm G (2016). Structural studies of lipopolysaccharide defective mutants from *Brucella melitensis* identify a core oligosaccharide critical in virulence. J Biol Chem.

[51] Conde-Álvarez R, Arce-Gorvel V, Gil-Ramírez Y, Iriarte M, Grilló MJ, Gorvel JP, Moriyón I (2013). Lipopolysaccharide as a target for brucellosis vaccine design. Microb Pathog.

[52] Moriyón I, Grilló MJ, Monreal D, González D, Marín C, López-Goñi I, Mainar-Jaime RC, Moreno E, Blasco JM (2004). Rough vaccines in animal brucellosis: structural and genetic basis and present status. Vet Res.

[53] Grilló MJ, Blasco JM, Gorvel JP, Moriyón I, Moreno E (2012). What have we learned from brucellosis in the mouse model?. Vet Res.

[54] Martirosyan A, Moreno E, Gorvel JP (2011). An evolutionary strategy for a stealthy intracellular *Brucella* pathogen. Immunol Rev.

[55] Porte F, Naroeni A, Ouahrani-Bettache S, Liautard JP (2003). Role of the *Brucella suis* lipopolysaccharide O antigen in phagosomal genesis and in inhibition of phagosome-lysosome fusion in murine macrophages. Infect Immun.

[56] Muñoz PM, Marín CM, Monreal D, Gonzalez D, Garin-Bastuji B, Diaz R, Mainar-Jaime RC, Moriyon I, Blasco JM (2005). Efficacy of several serological tests and antigens for diagnosis of bovine brucellosis in the presence of false-positive serological results due to *Yersinia enterocolitica* O:9. Clin Diagn Lab Immunol.

[57] Brinley Morgan WJ, Littlejohn AI, MacKinnon DJ, Lawson JR (1966). The degree of protection given by living vaccines against experimental infection with *Brucella melitensis* in goats. Bull World Health Org.

[58] Pérez-Sancho M, Adone R, García-Seco T, Tarantino M, Diez-Guerrier A, Drumo R, Francia M, Domínguez L, Pasquali P, Álvarez J (2014). Evaluation of the immunogenicity and safety of *Brucella melitensis* B115 vaccination in pregnant sheep. Vaccine.

