# Supplementary material for: A Brucella melitensis H38ΔwbkF rough mutant protects against Brucella ovis in rams
Source: Vet Res. 2022 Mar 2;53:16. doi: 10.1186/s13567-022-01034-z (PMC8889640; doi:10.1186/s13567-022-01034-z)
Supplement: Supplementary file 1 — Additional file 1: Primers used for mutant construction. [file 13567_2022_1034_MOESM1_ESM.docx]

**Additional file 1 Primers used for mutant construction**

| **Primers** | **Sequence 5´- 3´** | **Used to verify** | **Reference** |
| --- | --- | --- | --- |
| H38∆*wbkF* |  |  |  |
| *wbkF*-F1 | TGGCTACGCCCCAACAAATC´ | *wbkF* deletion | [[38](#_ENREF_1)] |
| *wbkF*-R4 | GACGCGCTATTTCATGTCCATC | *wbkF* deletion | [[38](#_ENREF_1)] |
| *wbkF-R6* | TTCATAGCGGTCATCCAGTCC | *wbkF* deletion | [[38](#_ENREF_1)] |
| Bov::CAΔ*wadB* |  |  |  |
| *wadB*-F1 | GCATGATTACCCCGCTGAT | *wadB* deletion | [[36](#_ENREF_2)] |
| *wadB*-R4 | TGATAGCCGAGCCTCTTCAG | *wadB* deletion | [[36](#_ENREF_2)] |
| *wadB*-R5 | ATGCACCCATGAAGTTTTCC | *wadB* deletion | [[36](#_ENREF_2)] |
| Rev1::*wbdR*Δ*wbkC* |  |  |  |
| *GlmS*_B | GTCCTTATGGGAACGGACGT | *wbdR* insertion | [[3](#_ENREF_3)9] |
| Ptn7-R | CACAGCATAACTGGACTGATT | *wbdR* insertion | [[3](#_ENREF_3)9] |
| Ptn7-L | ATTAGCTTACGACGCTACACCC | *wbdR* insertion | [[3](#_ENREF_3)9] |
| *RecG* | TATATTCTGGCGAGCGATCC | *wbdR* insertion | [[3](#_ENREF_3)9] |
| *Km*R-F1 | AGGAAGCGGAACACGTAGAA | *km* deletion | [[3](#_ENREF_3)9] |
| *Km*R-R4 | TGGTCCATATGAATATCCTCCTTA | *km* deletion | [[3](#_ENREF_3)9] |
| *wbkC*-F1 | AGGTGGCGACAAACGAATAA | *wbkC* deletion | [[3](#_ENREF_3)9] |
| *wbkC*-R4 | tctgaactcggctggatgac | *wbkC* deletion | [[3](#_ENREF_3)9] |
