# Supplementary material for: A Brucella melitensis H38ΔwbkF rough mutant protects against Brucella ovis in rams
Source: Vet Res. 2022 Mar 2;53:16. doi: 10.1186/s13567-022-01034-z (PMC8889640; doi:10.1186/s13567-022-01034-z)
Supplement: Supplementary file 2 — Additional file 2: E. coli S17 λpir and plasmids used for mutant construction. [file 13567_2022_1034_MOESM2_ESM.docx]

**Additional file 2 *E. coli* S17 λpir and plasmids used for mutant construction**

| **Strain/Plasmid** | **Characteristics** | **Reference** |
| --- | --- | --- |
| ***Escherichia coli*** |  |  |
| *E. coli* S17 λpir | Mating strain with plasmid RP4 inserted into the chromosome | [[37](#_ENREF_4)] |
| **Plasmids** |  |  |
| pJQKΔ*wadB* | *Bam*HI-*Xba*I fragment from pYRI-1 (containing 570 bp of *B. abortus* chromosomal DNA with the *wadB* deletion allele) cloned into the corresponding sites of pJQKm (Internal code pYRI-2) | [[36](#_ENREF_2)] |
| pJQKΔ*wbkF* | *Bam*HI-*Xba*I fragment from pRCI-16 (containing 953 bp of *B. melitensis* 16M chromosomal DNA containing the *wbkF* deletion allele) cloned into the corresponding sites of pJQKm (Internal code pRCI-17) | [[38](#_ENREF_1)] |
