# Supplementary figures and images for: A Brucella melitensis H38ΔwbkF rough mutant protects against Brucella ovis in rams
Source: Vet Res. 2022 Mar 2;53:16. doi: 10.1186/s13567-022-01034-z (PMC8889640; doi:10.1186/s13567-022-01034-z)

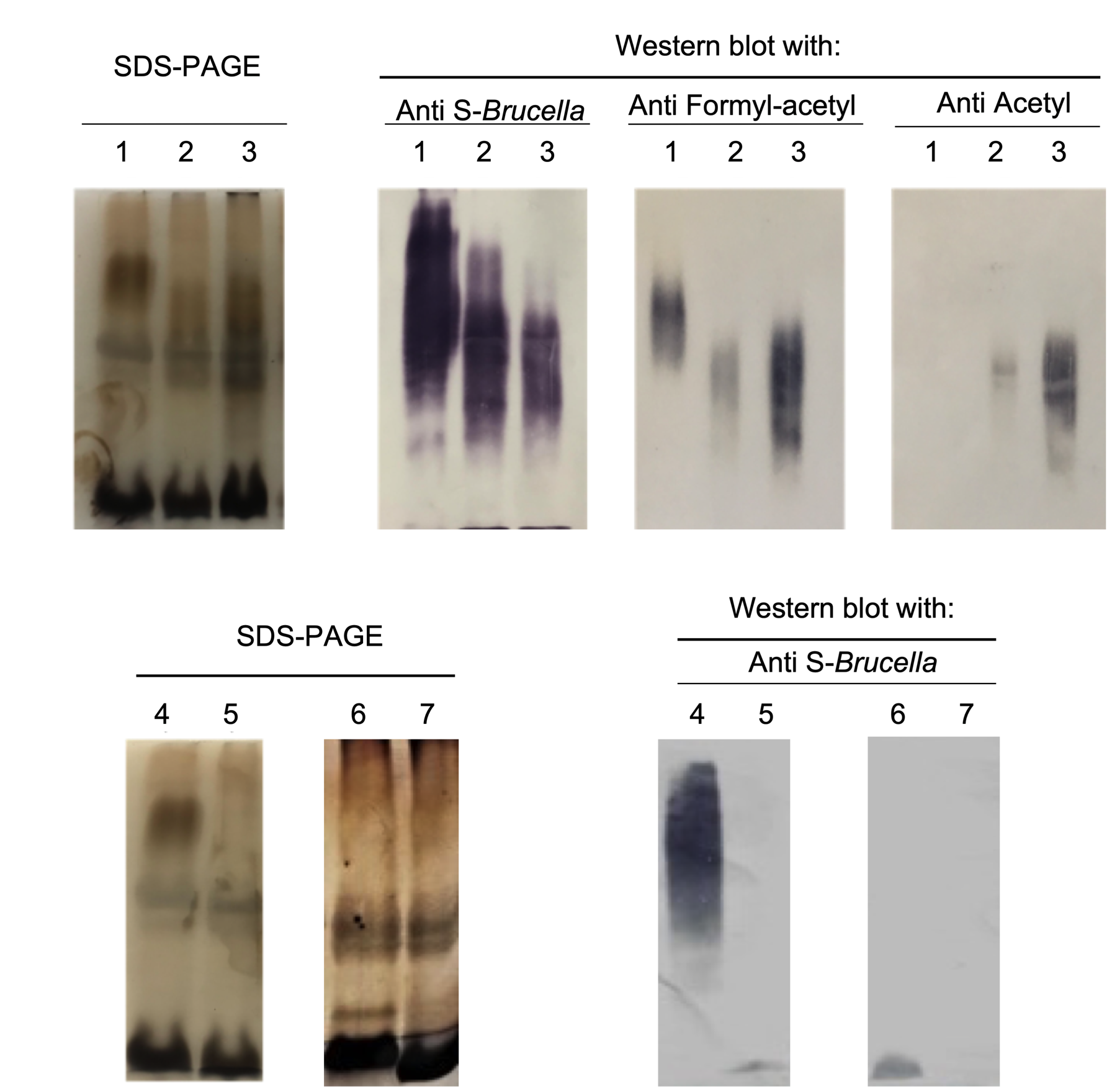

Supplement: Supplementary file 3 — Additional file 3: LPS characterization of vaccine candidates by SDS-PAGE electrophoresis-silver staining and Western blot analyses of SDS-proteinase K extracts. Upper panels (from [19]): Analyses of (1) Rev 1, (2) Rev1::Tn7wbdR, and (3) Rev1::Tn7wbdRΔwbkC extracts. Lower panels: Analyses of (4) H38, (5) H38ΔwbkF, (6) Bov::CA and (7) Bov::CAΔwadB extracts. [file 13567_2022_1034_MOESM3_ESM.tiff]
