# Supplementary material for: A Brucella melitensis H38ΔwbkF rough mutant protects against Brucella ovis in rams
Source: Vet Res. 2022 Mar 2;53:16. doi: 10.1186/s13567-022-01034-z (PMC8889640; doi:10.1186/s13567-022-01034-z)
Supplement: Supplementary file 4 — Additional file 4: Reactivity (%OD) in iELISA using N-formyl-perosamine wild-type S-LPS (A) or N-acetyl-perosamine S-LPS from a wbdRΔwbkC construct (B) and 46 sera from B. melitensis culture positive (C+) sheep, 78 brucellosis-free (BF) sheep and rams immunized with Rev1::wbdRΔwbkC. [file 13567_2022_1034_MOESM4_ESM.pptx]

## Slide 1
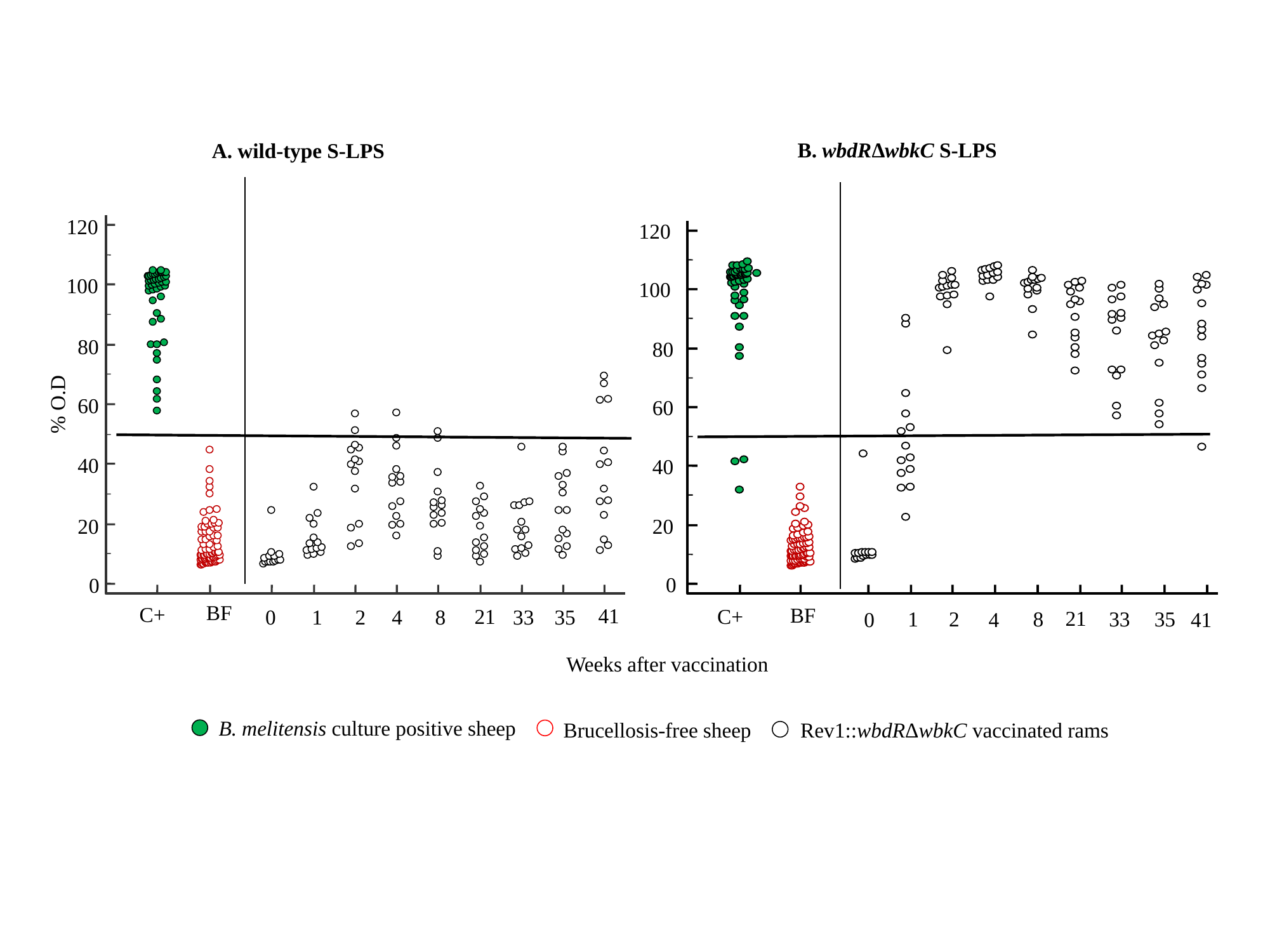

B. wbdRΔwbkC S-LPS
120
100
80
60
40
20
0
BF
C+
1
2
3
4
5
6
7
8
9
10
11
21
1
2
33
8
35
0
41
4
% O.D
A. wild-type S-LPS
120
100
80
60
40
20
0
BF
C+
41
21
1
2
3
4
5
6
7
8
9
10
11
1
2
33
35
8
0
4
Weeks after vaccination
B. melitensis culture positive sheep
Brucellosis-free sheep
Rev1::wbdRΔwbkC vaccinated rams
